# High surface-area carbon microcantilevers†

**DOI:** 10.1039/c8na00101d

**Published:** 2019-01-01

**Authors:** Steven G. Noyce, Richard R. Vanfleet, Harold G. Craighead, Robert C. Davis

**Affiliations:** Department of Physics and Astronomy, Brigham Young University Provo UT 84602 USA davis@byu.edu; School of Applied and Engineering Physics, Cornell University Ithaca NY 14853 USA

## Abstract

Microscale porous carbon mechanical resonators were formed using carbon nanotube templated microfabrication. These cantilever resonators exhibited nanoscale porosity resulting in a high surface area to volume ratio which could enable sensitive analyte detection in air. These resonators were shown to be mechanically robust and the porosity could be controllably varied resulting in densities from 10^2^ to 10^3^ kg m^−3^, with pore diameters on the order of hundreds of nanometers. Cantilevers with lengths ranging from 500 μm to 5 mm were clamped in a fixture for mechanical resonance testing where quality factors from 10^2^ to 10^3^ were observed at atmospheric pressure in air.

## Abstract 2

Fully-porous carbon resonators were formed with precise microscale dimension and characterized for sensitive detection of vapors.

## Graphical abstract

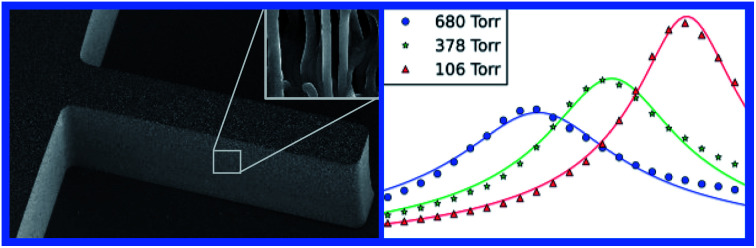

Realizing the vision of using chemical sensors to aid in broadly connecting the digital and physical worlds will require the capability to sense and analyze complex mixtures of molecules and to do so with low-cost miniaturized systems. Miniaturized chemical sensing could be enabled by arrays of microscale and nanoscale resonant cantilever sensors like those that have been developed to enable detection of analytes in air and other gas environments.^1–5^ In these systems, analyte binding to the cantilever surface can cause a variety of mechanical changes to the cantilever including adding mass, changing stiffness, mechanical loss, and static stress. The chemical selectivity of these sensors derives from the intrinsic chemical binding specificity of the sensor material or the binding specificity of a polymer layer or monolayer coating on the sensor.^2,3^

However, there is a general size-based trade-off that presents a challenge to improving cantilever detection sensitivity. Smaller cantilevers have a greater surface to volume ratio resulting in greater mechanical changes from surface analyte adsorption. Larger cantilevers are less affected by fluid damping, resulting in a sharper resonance (and higher quality factor) peaks and correspondingly better detection of resonance changes. Nanoporous cantilever resonators do not suffer from this trade-off and could thereby enable high sensitivity detection by significantly increasing the active surface area for analyte binding in geometries compatible with high resonance quality factors (see section on analysis of sensitivity and porosity).

Here we present the fabrication and characterization of high surface area carbon microcantilever resonators. These carbon nanotube/carbon composite cantilevers were made using the carbon nanotube templated microfabrication (CNT-M) process. The CNT-M process creates three dimensional shapes with micro-scale features from nanoporous material. The resulting structures can have aspect ratios of up to 200 : 1.^6,7^ The cantilever fabrication process is illustrated in Fig. 1 (see methods section below for details). Briefly, a thin film of iron was patterned on an alumina coated silicon substrate and carbon nanotubes were then grown perpendicular to the substrate from the patterned iron catalyst. The interstices between the nanotubes were partially infiltrated with a nanocrystalline carbon matrix material to form a cohesive structure.^8,9^ Precisely defined CNT-M cantilevers (see Fig. 2) were microfabricated with a wide range of porosities by varying the infiltration time.

**Fig. 1:**
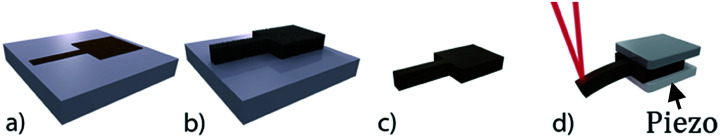
Microcantilever fabrication and measurement process consists of (a) photolithographic patterning of a 4 nm iron catalyst film, (b) vertical carbon nanotube growth from iron catalyst, (c) infiltration of the nanotube forest and release from the substrate, and (d) mounting of the device between a clamp and an AC voltage-driven piezoelectric element followed by deflection measurement by means of a reflected laser.

**Fig. 2:**
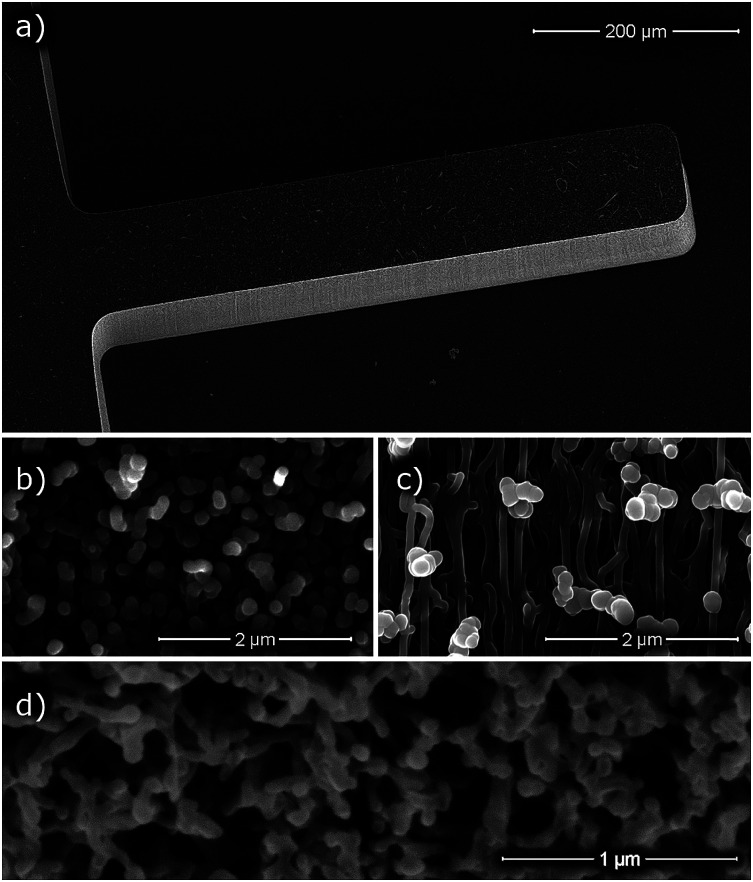
Scanning electron micrographs (SEM) of CNT-M microcantilevers. (a) a microcantilever extending from a larger base with the entire device resting on a silicon substrate, (b) microcantilever top surface, and (c) microcantilever side wall. (d) an SEM image of a CNT-M microcantilever that was cross sectioned by focused ion beam (FIB) prior to imaging. The samples in (a), (b), and (d) were infiltrated for 2 minutes, and (c) for 4 minutes.

Characterization of the resulting structures was performed to demonstrate the feasibility of these structures for development as a sensing platform. Specifically, CNT-M resonators were shown to be sufficiently robust for handling and integration into sensing setups. And it was demonstrated that the nanoscale porosity of the resonators can be varied and quantified over a large range. Tunable porosity is a functional property that can have significant impact on sensitivity, analyte capacity, diffusion times, and mechanical response. The ability to control and quantify porosity will be critical in developing sensors for specific applications using the CNT-M platform.

Additionally, a critical concern was answered relative to resonant loses in this porous platform. The concern was whether the resonant behavior would be dominated by internal resonance losses due to the large internal surface area. If the resonators were limited by large internal resonance losses, this could significantly limit applicability of this sensing platform for low detection limit sensing. To determine the relative contribution of fluid damping and other mechanical losses, the resonant characteristics of the CNT-M microcantilevers were measured at various pressures. In air the cantilevers had quality factors from 10^2^ to 10^3^ (at atmospheric pressure). Internal losses did not play a dominant role in damping: roughly half the damping came from fluid damping with the next largest source of loss likely being clamping losses.

The resonant frequency shift from exposure to water vapor was measured on uncoated (*i.e.* partially infiltrated with carbon but lacking any coating designed to increase analyte absorption or specificity) CNT-M cantilevers, resulting in 
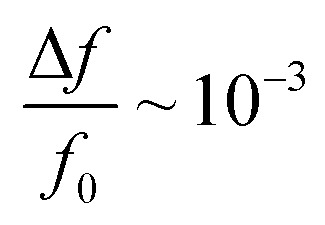
 at 100% relative humidity.

## Analysis of cantilever resonance sensitivity and impact of porosity

The added mass from an analyte bound to a cantilever is detected by monitoring changes in the cantilever resonance frequency. The added mass Δ*m* of a bound analyte causes a resonance frequency shift Δ*f* according the following relationship:1
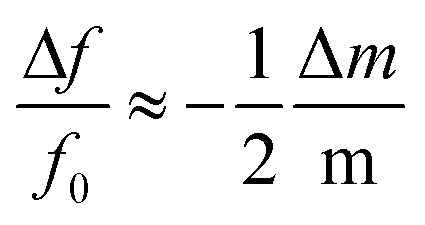
where *f*_0_ is the cantilever resonance without the added mass and *m* is the cantilever mass.

Sensitive analyte detection depends on several factors including the cantilever surface area, mass, and resonance quality factor. The added mass Δ*m* of a bound analyte is proportional to the product of the analyte concentration *C* and the cantilever surface area *S*. The mass of the cantilever depends linearly on the cantilever density *ρ* and cantilever volume *V*_c_. Putting these relationships into eqn (1) results in a resonance frequency shift of2
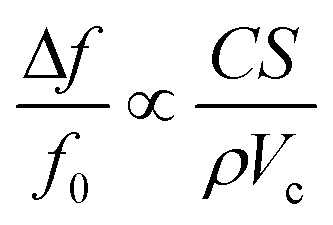


The concentration limit of detection *δC* also depends on minimum detectable frequency shift which is inversely proportional to the resonator quality factor *Q*.^5^ Putting this together yields a concentration limit of detection of3
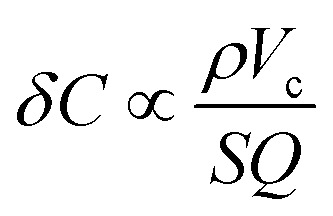


Utilizing a porous cantilever material will increase the available surface area and decrease the cantilever density resulting in a smaller (improved) limit of detection. Prior researchers have increased surface area by roughening the surface or adding a thin porous layer on the surface of a solid cantilever.^10–14^ Fully porous cantilever resonator and torsional resonators have been implemented using anodic alumina and porous silicon respectively.^15,16^

A fully porous cantilever sensor has the advantage that the ratio *S*/*V*_c_ is independent of the cantilever size or geometry (for *V*_c_ ≫ *V*_pore_, which is true across the range of sizes studied). In a solid cantilever, by contrast, increasing the volume by making it thicker reduces *S*/*V*_c_ (reducing Δ*m*/*m*). This presents a design tradeoff between having a high *Q* in a gas environment (less impact of fluid damping on a stiffer thick cantilever) and high sensitivity (from the higher *S*/*V*_c_ of a thin cantilever). In fully porous cantilevers this tradeoff does not happen as increasing cantilever thickness also increases *Q* but without lowering *S*/*V*_c_. This can be taken advantage of due to the flexibility of the CNT-M process to fabricate resonant structures in a wide variety of geometries.

## Results/Discussion

CNT-M cantilevers of various sizes were fabricated with lengths ranging from 500 μm to 5 mm with a thickness of ∼200 μm. The cantilevers were imaged by scanning electron microscope (a representative image is shown in Fig. 2), showing the overall cantilever structure and revealing the porosity. From Fig. 2a we can see that the geometry of the cantilever is well defined: the catalyst pattern precisely controls the top-down shape, uniform CNT forest growth yields a uniform height and gives relatively vertical sidewalls. The top surface, sidewall, and focused ion beam (FIB) cross-sectioned images seen in Fig. 2b–d indicate that the structure is porous. The FIB milled cross sectional view gives an indication of the distance between nanotubes and of the coated nanotube diameters, but further investigation was required to more quantitatively measure these.

Due to ambiguity in differentiating the FIB cut CNT ends from other features near the cut surface (as seen in Fig. 2d), we further prepared some CNT-M microcantilevers for quantitative analysis of the nanotube number density. This was done by infiltrating the cantilevers with a contrasting material prior to preparing FIB-milled cross-sections for analysis. We explored infiltration with two different contrast materials for this purpose, epoxy and electroplated nickel. Nickel filled samples were fabricated using a previously developed electroplating process^8^ described in the ESI.† Nickel filled samples imaged well, producing good contrast (see ESI† for sample image of CNT-M structures with nickel contrast infiltration). Epoxy filled samples were more difficult to image in the SEM due to sample charging, but could still be used to analyze CNT density. Analyzing the SEM images yielded areal density numbers of 89–152 nanotubes per μm^2^ and 70–139 nanotubes per μm^2^ for iron catalyst thicknesses of 4 nm and 7 nm respectively. Using a hexagonal packing model, these densities correspond to mean distances between nearest neighbors of 87–114 nm for the 4 nm catalyst and 91–128 nm for the 7 nm catalyst.

**Fig. 3:**
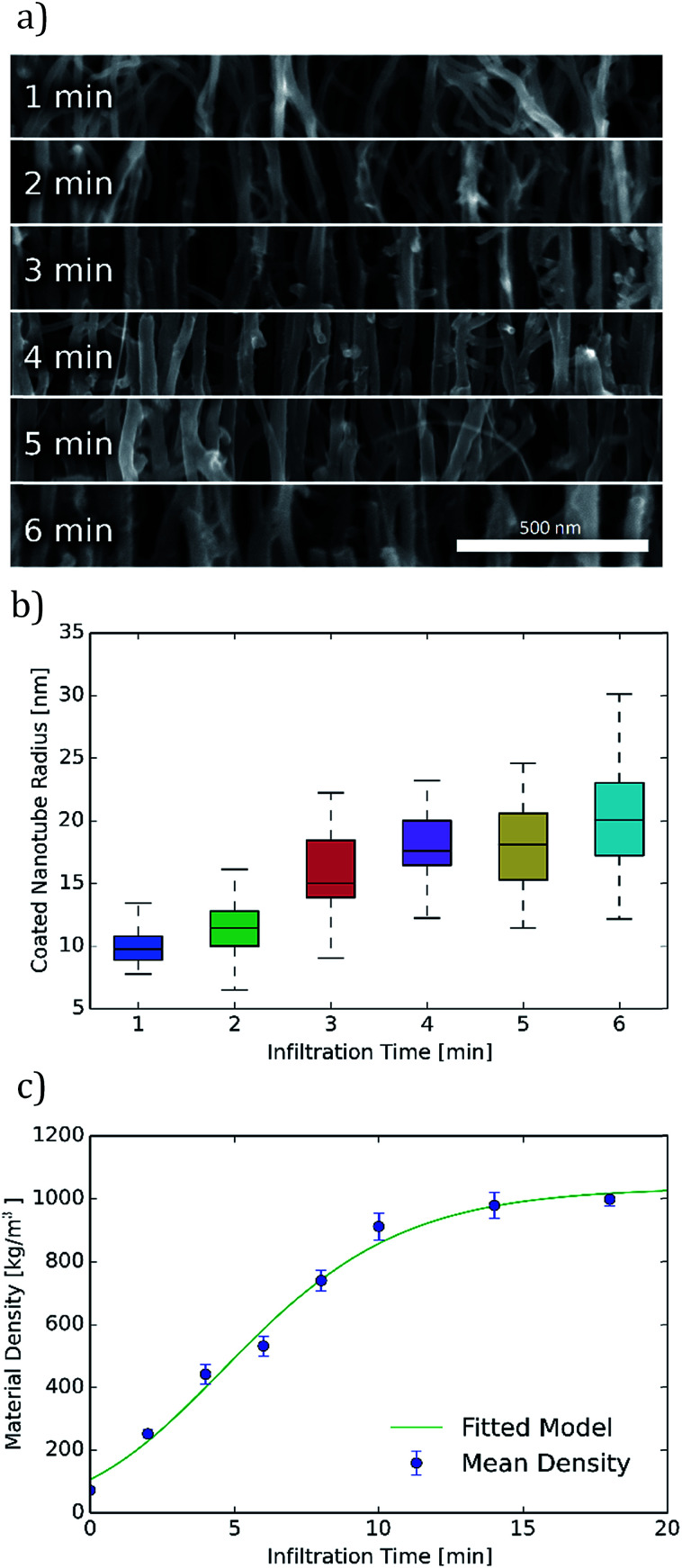
Quantitative measurements of infiltrated carbon nanotube forest porosity. (a) Coated carbon nanotube sizes at infiltration times of 1 to 6 minutes as indicated in the image. (b) Coating radius *vs.* infiltration time. Cylindrical radius of coated carbon nanotubes is shown for various infiltration times. (c) Bulk density for various infiltration times is shown along with a model of the coating process. Error bar extends below and above the mean by one standard deviation of the measured data and includes error estimates in measured mass and height along with sample to sample variation.

Carbon coated CNT diameters could not be measured directly from FIB milled samples due to additional carbon deposition during the milling process which obscured the sample diameters. Therefore, for diameter analysis, samples were mechanically broken prior to the SEM cross-section imaging shown in Fig. 3a. Analysis of these images indicates an increase in the diameter of coated carbon nanotubes with increasing infiltration time. Diameter measurements on 100 coated CNTs for each of these infiltration times from these and other similar images are summarized in Fig. 3b. The mean radius of coated nanotubes increases from approximately 10 nm after 1 minute of infiltration to over 20 nm after 6 minutes of infiltration. This radius continues to increase with longer infiltration times, but these shorter infiltration times comprise the range of greatest interest due to the high porosities obtained in this region.

Macroscopic density was measured for a wide range of infiltration times by weighing CNT-M structures of known dimension. These bulk density measurements show good agreement (within 15%) with densities calculated from nanotube number density and radius (for the overlapping infiltration times from 1 to 6 minutes). As seen in Fig. 3c, the structure reaches half of its maximal density after approximately 6 minutes of infiltration. The CNT-M process produces cantilevers with porosities that can be tuned over a wide range. Between 0 and 18 minutes of infiltration, density changed by over an order of magnitude, yielding densities from below 100 kg m^−3^ to above 1000 kg m^−3^ respectively. This corresponds to void fractions ranging from 8% to 92%. Infiltration times longer than about 6 minutes, while yielding smaller average pore sizes, will result in a structure with a void fraction less than about 50% and a less desirable surface to volume ratio for sensing. When smaller pores are desired, higher void fractions could be achieved by starting with a CNT growth process tuned for smaller CNT spacings.^17^

Resonance testing was performed on cantilevers with lengths ranging from 500 μm to 5 mm and thicknesses ranging from 50–500 microns. As illustrated in Fig. 1d, the cantilevers under test were mounted between a clamp and an AC voltage-driven piezoelectric element. The vertical movement of the cantilever was detected by monitoring the deflection of a laser reflected off the cantilever surface. The resonance responses were obtained by scanning the frequency of the AC voltage applied to the piezoelectric driver and measuring the AC response in the laser deflection signal. The cantilevers were found to have fundamental mode resonant frequencies from about 1 kHz up to 100 kHz.

**Fig. 4:**
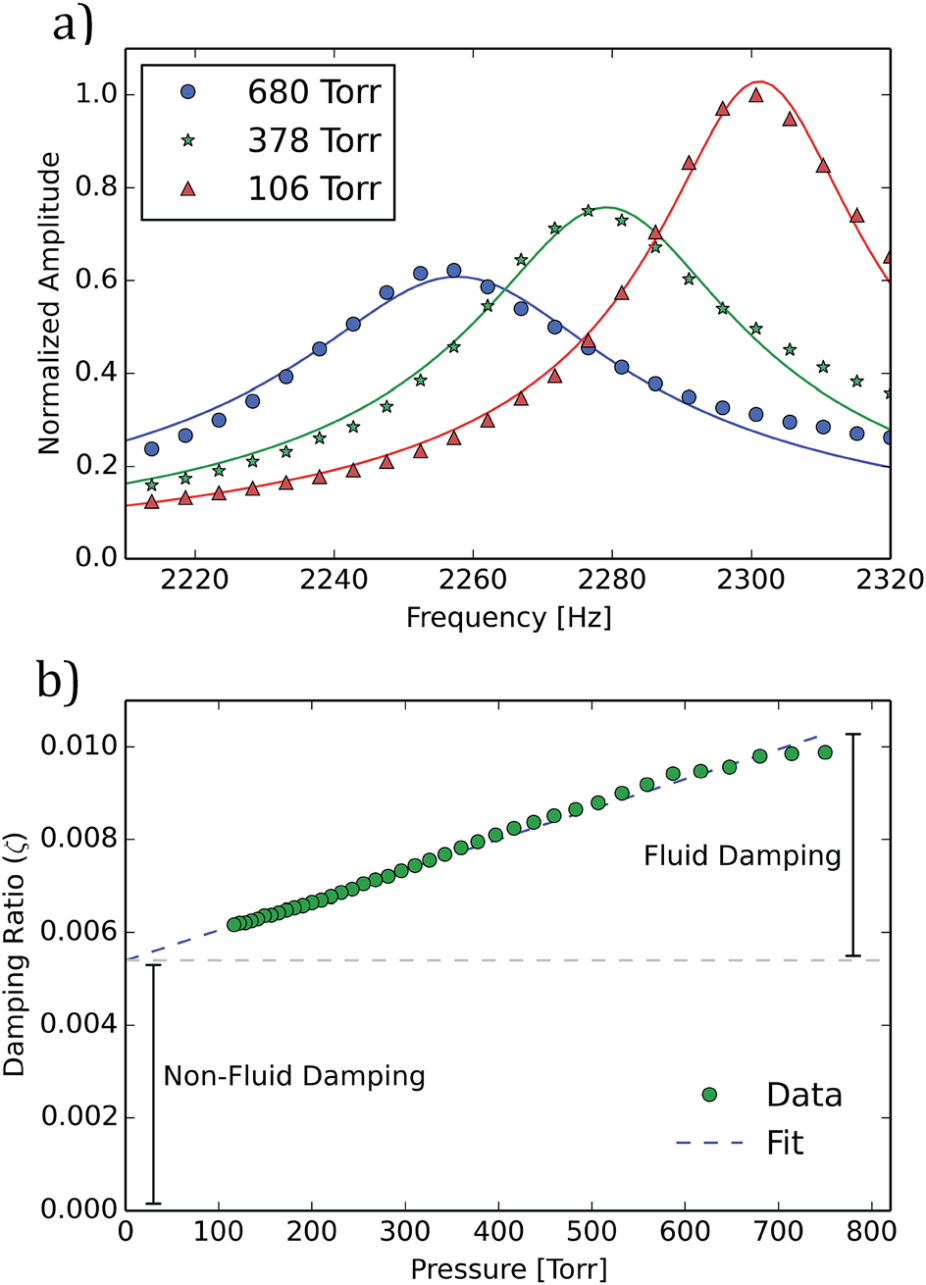
Resonance frequency and quality factor at various pressures. (a) Resonance data of a single cantilever (with dimensions of 4.3 mm long, 1 mm wide, ∼200 microns thick, and infiltrated for 10 min) in three partial air environmental pressures. Each data set is fit to a Lorentzian model shown alongside a model fitted to each case that was used to extract the resonance frequency and quality factor. (b) The value of the damping ratio (*ζ* = 1/(2*Q*)) for a given cantilever is shown for a range of environmental gas pressures. It can be seen that at atmospheric pressure approximately half of the total damping is due to fluid damping, the other half being due to clamping and other losses.

In addition to high surface areas and low volumetric densities, improved limits of analyte detection are enabled by resonators with high quality-factors(*Q*). Observed *Q* for these cantilevers ranged from approximately 10^2^ to 10^3^ in ambient air. *Q* and resonance frequency were determined by fitting a simple harmonic oscillator model to the measured resonance data with *f* and *Q* as the fitting parameters. Example fits are shown in Fig. 4a. The following damping mechanisms can contribute to the quality factor of the cantilever: fluid damping in air, clamping losses, and thermoelastic and surface loses specific to the porous material.

To examine the impact of air on the cantilever resonance frequency and quality factor, cantilever resonance was measured in a partial air vacuum with pressures down to 100 torr (see Fig. 4). As pressure decreases, both the resonance frequency and quality factor increase. This can be observed in the peaks which shift to the right and become taller and sharper as pressure drops (Fig. 4a). To analyze damping more directly, the damping ratio *ζ* (the ratio of measured damping to critical damping) was computed from the measured quality factor as *ζ* = 1/(2*Q*) and can be seen plotted against pressure in Fig. 4b. The damping ratio appears to follow a linear trend over the range of pressures studied. Fitting a linear model to the damping ratio data and evaluating that model at zero pressure gives an estimate of the amount of damping that comes from sources other than the fluid, such as clamping and materials losses. The data indicates that fluid damping in ambient air was as large as all other sources of damping combined. This relationship holds for the range of cantilever devices studied.

It is likely that a major contribution to non-fluid damping is the result of anchor/clamping losses due to the clamping setup. Mechanical clamping of the cantilevers was performed after the cantilevers were released (during fabrication) from the growth substrate prior to dynamic testing. Both the precision of the alignment of the clamp to the end of the beam as well as the clamping pressure were difficult to control and assess. The assertion that clamping loss is a major contribution to damping is strengthened by variations in *Q* that were seen when the same cantilever was re-clamped and re-measured.

Modeling and characterization of damping mechanisms with silicon cantilevers has contributed understanding that allows for optimization of dimensions resulting in a reduction of overall damping.^18^ While this prior analysis cannot be directly applied to the present CNT-M cantilevers due to significant differences in dimensions and modulus (which impact the assumption of those models) the damping observed in air with solid silicon cantilevers is in the same range of 10^2^ to 10^3^.

We expect that non-fluid losses, both those due to clamping losses and thermoelastic damping, could be significantly reduced in future CNT-M resonator designs through the use of narrow anchors placed at vibrational antinodes.^19^ There is also significant room to reduce fluid damping further through the design and fabrication of CNT-M cantilevers with different geometries including thicker cantilevers. Whereas these cantilevers were grown to heights of ∼200 μm, the CNT-M process has recently been used to fabricate structures with growth heights >1 mm.^20^

**Fig. 5:**
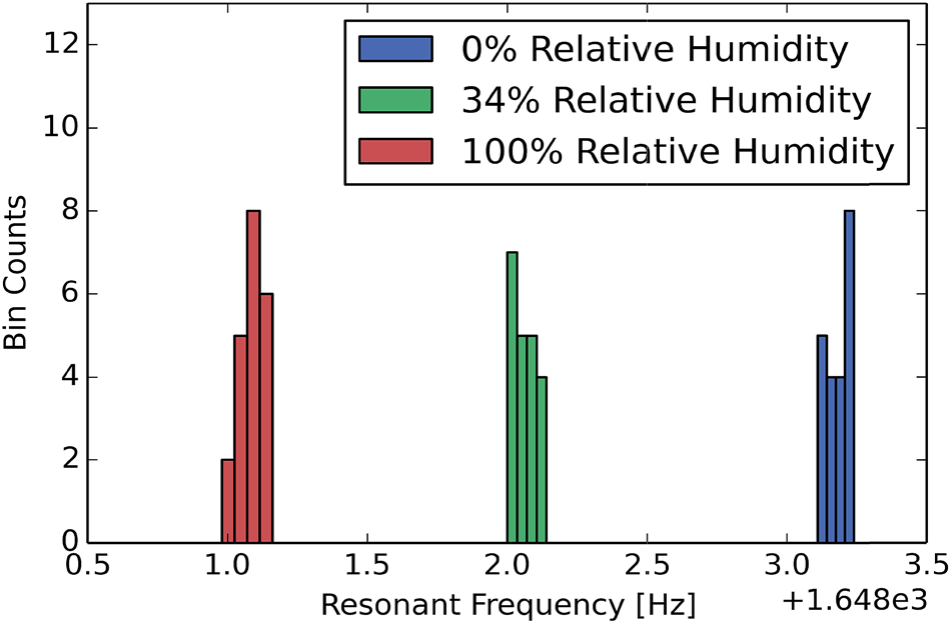
Resonant frequency of a cantilever is shown for relative humidity values of 0, 34, and 100 percent (cantilever dimensions are: 3 mm long, 100 μm wide, 64 μm high, and a carbon infiltration time of 6 minutes). This histogram shows observations taken while humidity values were sequentially changed in a randomized order. Shifts on the order of 1 Hz were seen on top of a base resonant frequency of 1.65 kHz.

To measure the sensitivity to vapor in air, the CNT-M microcantilevers were exposed to water vapor at relative humidity values of 0, 34, and 100 percent and the resulting resonant frequencies are displayed in Fig. 5. Although the adsorbed water from the environment could result in both modulus and mass change to the cantilever, we expect the observed shift is dominated by mass added. At 100% relative humidity, the shift resulted in 
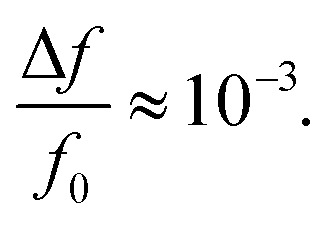


Although these CNT cantilevers have a much lower bulk modulus and are much thicker than silicon microcantilevers reported in the sensing literature a comparison of the frequency shift is still relevant. The data in Fig. 5 shows a resonant frequency shift greater than 0.1% in an uncoated 64 μm thick CNT cantilever exposed to a relative humidity of ∼30% water vapor. This response is slightly larger than that seen in the coated micro-cantilevers reported by Battiston *et al.*^3^ (where the largest shift was 0.05% under ∼30% water vapor exposure). These coated silicon cantilevers were 8 microns thick and were coated with a 2–3 micron thick polymer layer.

Because the current CNT-M cantilever was uncoated and hydrophobic, there remains a significant potential sensitivity gain by coating the CNTs with an appropriate polymer surface layer. Also, polymer layers or other chemically-selective coatings could be used to impart chemical discrimination. The addition of a polymer layer to the surface of the carbon coated CNTs is feasible, though use of techniques compatible with porous material coating are required; for example thin polymer coatings have previously been applied to CNT networks with similar nanoscale porosity using layer by layer assembly or electrodeposition.^21–23^ The conductivity of the CNT-M structure could enable multiplexed electrodeposition for array-based sensing as would be employed in an “electronic nose”.

## Conclusions

We have fabricated high surface-area carbon microcantilever mechanical resonators using the CNT-M process. The CNT-M microresonators were mechanically robust over a wide range of controllable porosities, providing a promising platform for sensitive analyte detection in air or gas environments.

Nanoscale porosity was controllably varied and characterized for various carbon infiltration times. We were able to determine coated CNT diameters, CNT bulk density, and CNT number areal densities. In addition to the microresonators described here, this characterization will also be valuable for other applications of the CNT-M process.

The quality factors in air and resonant shifts under exposure to water vapor of these initial CNT-M cantilevers were characterized. The porous CNT-M cantilever resonators had quality factors in air ranging from 10^2^ to 10^3^ which is comparable to that of solid microcantilevers in air. Internal losses do not appear to dominate cantilever damping. Fluid damping accounted for about 50% of the damping with the other 50% of the damping coming most likely from clamping due to limitations in the setup. A large frequency shift in response to water vapor was seen at several vapor concentrations; the shift was comparable to published results for solid cantilevers coated with polymer layers, resulting in 
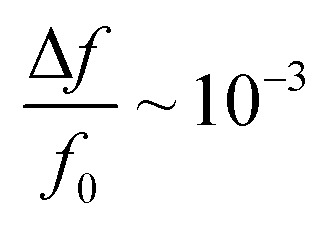
 at 100% relative humidity.

The limits of detection for resonant detectors improves with both increasing frequency shift sensitivity and increasing quality factor. There appears to be room for significant additional gain in both frequency shift sensitivity and quality factor for the CNT cantilevers. Coating the CNT-M structures with thin polymers should result in significantly higher concentration dependent frequency shift sensitivities while providing chemical specificity. The fully porous nature of the cantilevers and the versatility of the CNT-M process allows significant design latitude for further reductions in fluid damping, clamping loss, and thermoelastic damping, by changing the size and shape of the resonator. These gains in frequency shift sensitivity and the increased *Q* through optimized mechanical designs could result in improvements in the limit of detection by greater than a factor of 10.

## Experimental

### Cantilever fabrication

The microcantilever fabrication procedure is an implementation of the CNT-M process, and is illustrated in Fig. 1. First, 30 nm of alumina was deposited onto a silicon wafer by electron beam evaporation, then a 4 nm thick micropatterned iron film was applied by contact photolithography, thermal evaporation, and liftoff. This iron film serves as the catalyst for carbon nanotube growth.

Next, the sample was placed into a 1-inch diameter tube furnace with 230 sccm (standard cubic centimeters per minute) of hydrogen gas flowing over the sample as it was heated to 750 °C. This hydrogen flow reduces any oxidation of the iron film. When the furnace arrives at the indicated temperature, carbon nanotubes were grown by adding 230 sccm of ethylene gas to the flow for the desired growth time. Growth times ranged from ∼1 to 10 minutes resulting in growth heights ranging from about 50 μm to 500 μm.

The flow of hydrogen and ethylene was then replaced with an argon flow to flush the chamber with an inert atmosphere while the furnace was ramped up to 900 °C. The carbon nanotubes were then infiltrated with nanocrystalline carbon^24^ by replacing the argon flow with the same flow rates of hydrogen and ethylene as used previously for carbon nanotube growth. At the end of the desired infiltration time (ranging from 1–15 minutes) the gas flows were again replaced with argon flow as the furnace was cooled to room temperature.

Cantilevers were released from the silicon substrate by immersing in 45% wt KOH for 4 hours at room temperature. Some of the cantilevers that were infiltrated for longer times self-released from the substrate upon cooling after infiltration, eliminating the need for the KOH step. Following release, the cantilever structure was placed in a clamping mechanism for testing as indicated in Fig. 1d for resonance testing. The clamping mechanism was comprised of two metal plates, fastened together using small screws. The cantilever and piezoelectric are placed between these two plates before fastening.

### Porosity and nanostructure

To explore material porosity and nanostructure, devices were fabricated with a range of infiltration times ranging from 1 to 15 minutes. The effective density of the structures was computed from their dimensions and mass. For each fabricated device, the maximum growth height was measured with a micrometer and the mass of the device was obtained by use of a microbalance (device masses were on the order of 5 mg). The cross-sectional area of each device was defined through photolithography.

The diameter of coated nanotubes was determined by breaking a sample along a plane parallel to the nanotube growth direction and imaging the exposed coated nanotubes by SEM. These fracture planes are imaged at the bottom, midpoint, top, and side of the nanotube forests to determine coating uniformity. From each of these images, the diameters of one hundred nanotubes in the focal plane are measured. The radius was also determined as half of the measured diameter.

The area number density of nanotubes in a cross-section perpendicular to the growth direction was measured by three different methods. First, cross sections of the as fabricated devices were exposed by milling with a focused ion beam (FIB), then imaged *via* SEM. Second, as fabricated devices were first infiltrated with epoxy resin (M-Bond) before a cross section was exposed by mechanical polishing and imaged by SEM. Third, as fabricated devices were first infiltrated with electroplated nickel (see ESI† for description of nickel electroplating method and resulting SEM image) to provide a good conductive path for SEM imaging before a cross section was milled by FIB and imaged with SEM.

### Resonance testing

To measure resonant frequency and resonance quality factor, the piezoelectric was driven with an AC voltage. To determine the amplitude of the cantilever motion, a laser was directed at the cantilever tip such that the tip reflects a portion of the beam. A photodiode was then placed to receive the reflected beam, with roughly half the reflected light falling on the photodiode. This arrangement yields a modulation on the photodiode signal (monitored with a lock-in amplifier) as the cantilever oscillates.

## Conflicts of interest

There are no conflicts to declare.

## Supplementary Material

NA-001-C8NA00101D-s001
