# Supplementary material for: High surface-area carbon microcantilevers
Source: Nanoscale Adv. 2019 Jan 1;1(3):1148–54. doi: 10.1039/c8na00101d (PMC9418787; doi:10.1039/c8na00101d)
Supplement: NA-001-C8NA00101D-s001 [file NA-001-C8NA00101D-s001.pdf]

Electronic Supplementary Material (ESI) for Nanoscale Advances.
This journal is © The Royal Society of Chemistry 2018

# SUPPLEMENTAL INFORMATION

## Nickel Electroplating and FIB milling to create cross sections for density measurements.

The process used to plate nickel into the interstices between carbon nanotubes in a patterned structure such as a cantilever is described in Reference [14]. Briefly, it consists of:

1. Exposing CNT forests to ozone for 30 minutes.
2. Preparing a plating solution of 12.5 grams of Nickel Chloride Hexahydrate, 1.25 grams Boric Acid, and 100 ml of Deionized water, and stirring with a magnetic stirring rod for 30 minutes.
3. Attaching electrodes to nickel source and sample, then lowering both into the plating solution, heated to 50° C and actively stirred.
4. Performing pulse electroplating with repeated cycles of 8 A/cm$^3$ for 3 ms followed by no current for 27 ms.
5. Allowing plating to continue for ~8 hours, then removing, rinsing, and drying sample.
6. Gallium ion FIB milling of the nickel filled sample to expose a cross section the nickel infiltrated carbon nanotube sample.

An example cross section of the result is shown in Figure S1.

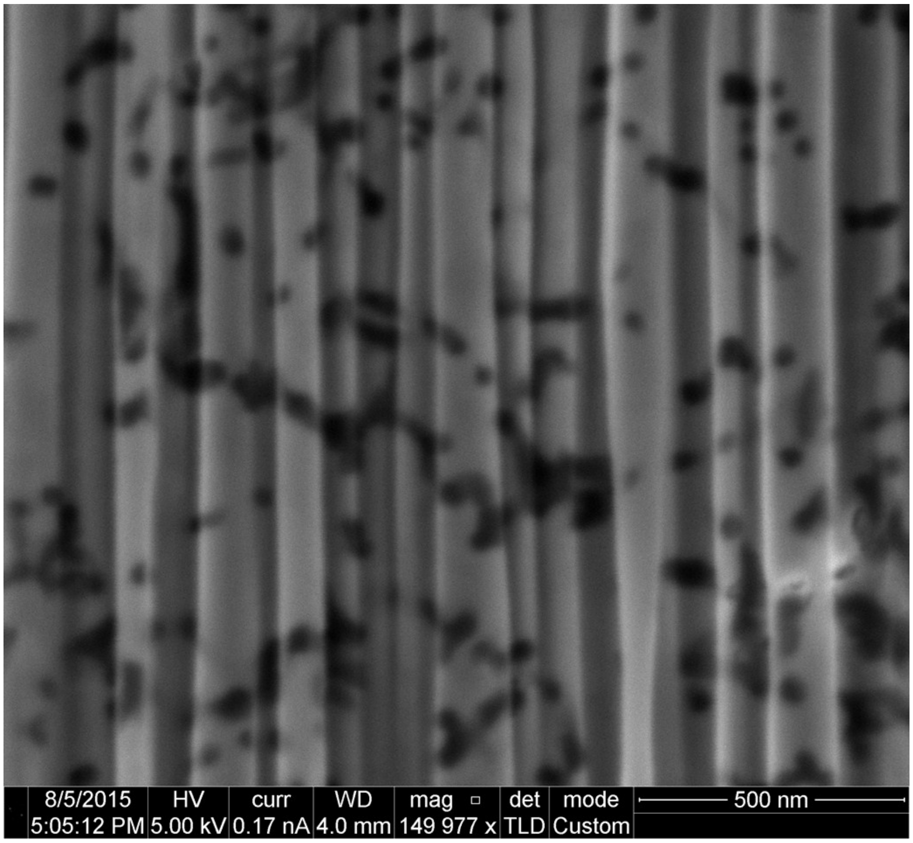


*Figure S 1* - A FIB cut cross section of a cantilever grown with 7 nm thick iron catalyst and infiltrated with nickel by pulsed electroplating. Although there is "curtaining" in the ion milling causing the undulation seen on the cross section the surface, density measurements can still be obtained with these images.

## Coating Radius versus Vertical Location in Structure

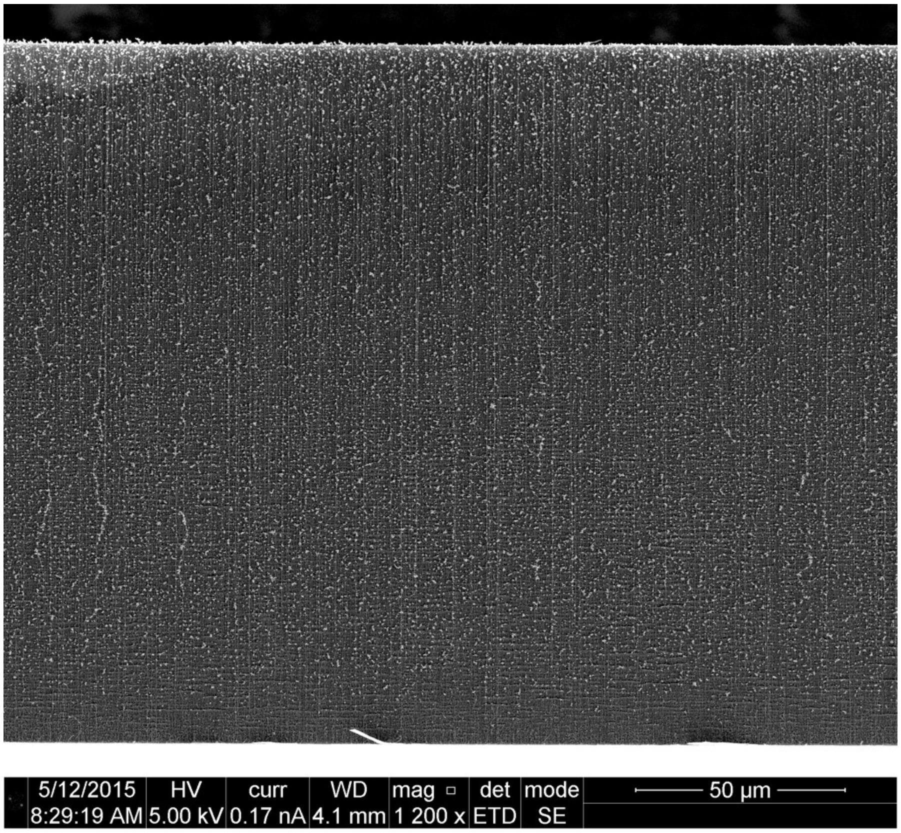


*Figure S 2* - A full exterior side wall shown from top to bottom of a ~200 µm thick cantilever grown from a 4 nm iron catalyst and infiltrated for 4 minutes. The density of coating nodules is lower near the bottom of the carbon nanotube forest, but the coating diameter is similar throughout the majority of the CNT length on these external walls.

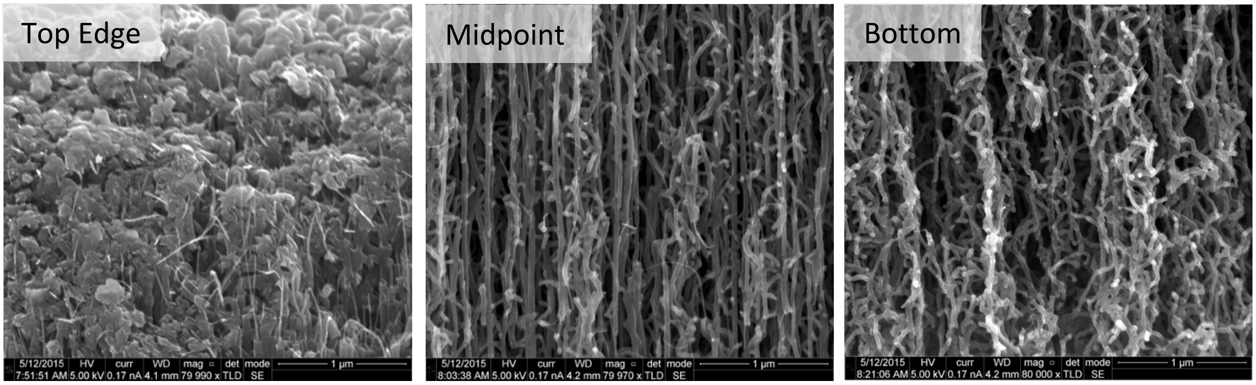


*Figure S 3 – Cleaved cross sectional images taken by SEM after a 5-minute infiltration. A thicker coating shell (capping layer) is formed along the top few microns of the forest, then the coating is more uniform with the coating diameter decreasing by 10-20% near the bottom few microns of the forest,*

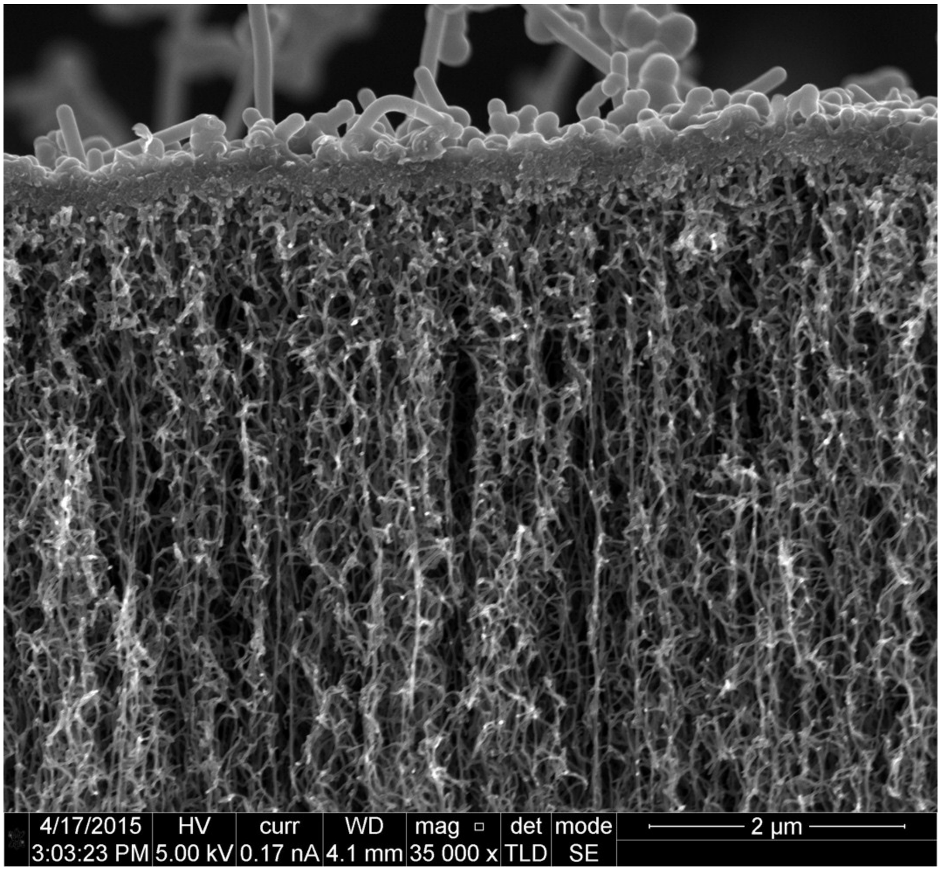


*Figure S 4 – Cleaved cross section of sample infiltrated for 2 minutes. Capping layer is apparent and distinct from the main body of the structure.*

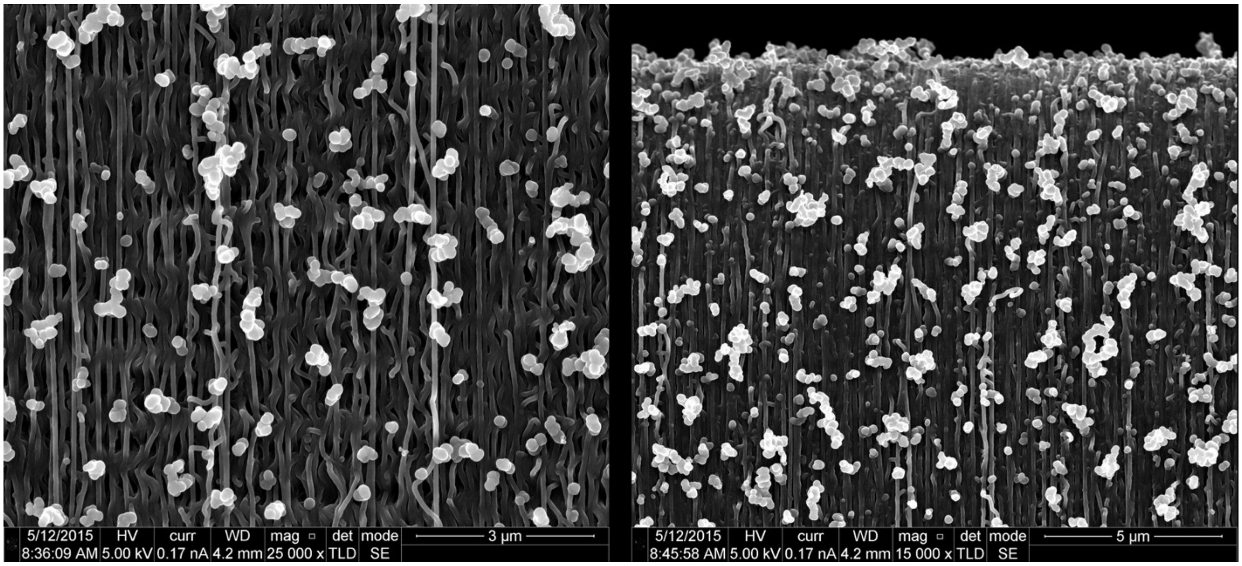


*Figure S 5 – Exterior sidewall of a sample with 4 minutes of infiltration. Two images showing the middle (left) and upper (right) sidewall of the same device. Qualitatively these images suggest that there may be more sidewall capping near the top of a device infiltrated for 4 minutes.*

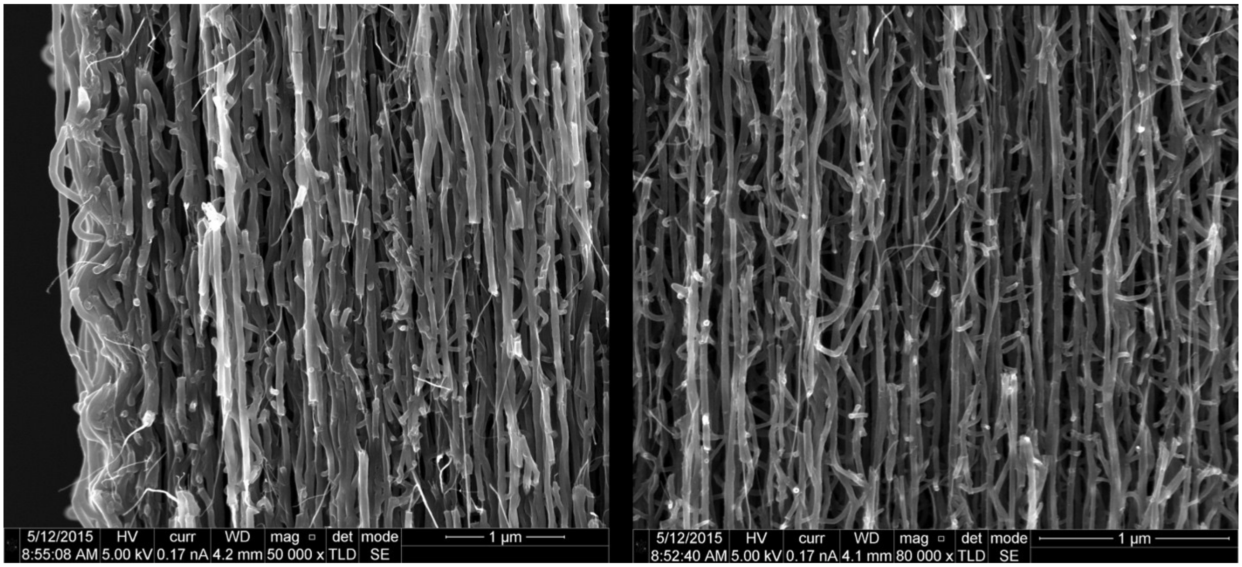


*Figure S 6 – Cleaved cross section of sample infiltrated for 4 minutes. Coated nanotubes near the device edge (left) have slightly larger diameters than those near the device center (right), and a small amount of sidewall capping is visible.*

Extraction of Resonance Frequencies and Quality Factors

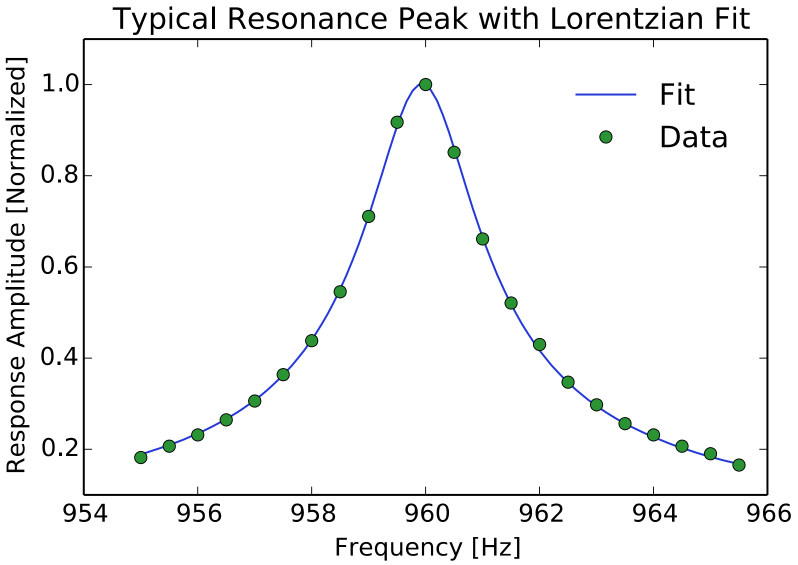


*Figure S 7 - An example result of fitting a simple harmonic oscillator model to resonance data in order to extract resonance frequency and quality factor.*
